# South West Surgical Club Autumn Meeting 1992

**Published:** 1992-12

**Authors:** 


### South West Surgical Club

### Abstracts of papers given at the Autumn Meeting held at the Royal United Hospital Bath October 23rd and 24th 1992

### COLOUR DUPLEX SCANNING IN THE INVESTIGATION OF PERIPHERAL VASCULAR DISEASE

T. S. Bates, M. Horrocks
Vascular Studies Unit
Royal United Hospital, Bath

Non-invasive assessment of lower limb arterial disease may be used to select those cases which would be amenable to angioplasty.

Fifty-four limbs from 40 patients presenting with claudication or critical ischaemia were studied prospectively. Intra-arterial DSA and colour duplex scanning (HDI, ATL) of the femoral and popliteal arteries was performed on all limbs. The iliac vessels were scanned in 12 limbs.

In the superficial femoral artery, 28 of 29 occlusions were correctly diagnosed and 19 of 21 stenoses. In the iliac vessels 8 out of 9 significant lesions were correctly detected. In every case the duplex scan was able to predict correctly whether the lesion would be suitable for angioplasty.

Duplex scanning is a highly accurate, non-invasive means of diagnosing the site and nature of vascular lesions, predicting those patients who would be appropriate for angioplasty. This would reduce the number of patients undergoing unnecessary angiography with its inherent risks and allow for more efficient usage of the angiography suite.

### TRANSCRANIAL DOPPLER INVESTIGATIONS IN PATIENTS UNDERGOING CAROTID ENDARTERECTOMY

T. R. Magee, A. H. Davies, R. N. Baird, M Horrocks
Vascular Studies Units
Bristol Royal Infirmary and the Royal United Hospital, Bath

Doppler techniques have largely taken over in the preoperative investigations and intraoperative monitoring of patients undergoing carotid endarterectomy (CEA). Duplex scanning is sufficient for the assessment of stenosis prior to surgery except where occlusion or subocclusion is suspected. Fifty five patients have been investigated using transcranial Doppler (TCD) before and during CEA. In addition stump pressure and internal carotid artery flow (qICA) was measured.

There was no correlation between stump pressure and change in middle cerebral artery velocity (Vmca) at the time of clamping (r <0.248). qICA was significantly lower in patients with >90% stenosis but maintained a mean flow of 137 ml/min. There was no significant difference in the fall in Vmca at clamping for patients with different degrees of stenosis.

Stump pressure is a poor predictor of Vmca changes due to clamping. Flow through a >90% stenosis contributes significantly to cerebral perfusion. Stump pressure and degree of stenosis should be used with caution in determining the need for intraoperative shunting.

### ANGIOSCOPY: A NEW TOY?

T. R. Magee, A. H. Davies, R. N. Baird, P. M. Lamont, M. Horrocks
Vascular Studies Units, Bristol Royal Infirmary and Royal United Hospital, Bath.

Angioscopy is becoming a popular new tool in vascular surgery but after initial enthusiasm will it find a place in routine work? The initial combined experience of the BRI and the RUH of using angioscopy for vein preparation in femoro-popliteal has been collated. Twenty patients have been studied, all have had in situ vein femoro-popliteal bypass for critical ischaemia. The procedure was performed using the standard groin and popliteal approaches but valve lysis was undertaken under angioscopic control. Side branches were identified by the scope and small incisions made to allow branches to be ligated.

The initial results are favourable, hospital stay was significantly shorter in these patients when compared with matched historical controls.

Further work will be undertaken in the two centres to further evaluate this "minimally invasive" technique but from our initial experience angioscopy is not just a new toy.

### RUPTURED ABDOMINAL AORTIC ANEURYSMS — ARE THEY SAFE TO TRANSFER?

M. S. Whiteley, C. Irvine, H. A. Everitt, A. Hinchliffe, R. N. Baird, H. Horrocks

Weston General Hospital (W.G.H.) is a district general hospital without a vascular surgical service. It is twenty six miles from the Bristol Royal Infirmary (B.R.I.), a teaching hospital with a vascular surgical department. In 1988, a decision was made in Weston General Hospital that all ruptured Abdominal Aortic Aneurysms would, after appropriate resuscitation, be transferred for treatment at the Bristol Royal Infirmary. We have studied the patients presenting to Weston General Hospital between February 1988 and July 1991 with a final diagnosis of ruptured abdominal aortic aneurysm and compared these to patients presenting directly to the Bristol Royal Infirmary. There was no significant difference between the ages, delay in presentation after onset of symptoms, nor length of operation in either group.

| | Age | Delay in Presentation | Time of Operation |
|---|---|---|---|
| **W.G.H. Patients:** | 72.7 (58-94) | 15.2 hours | 3.0 hours |
| **B.R.I. Patients:** | 73.9 (54-94) | 14.5 hours | 2.9 hours |

Of those patients arriving alive at each hospital, 33% of 36 patients from Weston General Hospital were discharged alive, despite the 26 mile transfer, the same proportion (33% of 61 patients) as those presenting directly to Bristol.

### ABSTRACT FOR SUBMISSION TO SOUTH WEST SURGEONS

**Surgical Goitres in Childhood and Adolescence : Cytological Aspects and Problems in Management**

A. John Webb
Consultant Surgeon
Bristol Royal Infirmary

Because of the rarity of goitres in childhood and adolescence (0-16 years inclusive), especially malignant lesions[1], there are difficulties in diagnosis and management. An exceptional papillary carcinoma arising in an 8 year old boy prompted a review of surgical goitres from the Bristol Childrens' Hospital and Bristol Royal Infirmary.

A 23 year span, from 1967-1989 inclusive, was studied using retrospective data, namely operative theatre records, histopathology and cytology files. The series was first presented in 1990 and has now been updated prospectively for 1990 and 1991. All histopathological (16) and cytological (8) material has been reviewed and re-evaluated.

The yield of patients was small, only 16 investigated and surgically treated goitres, (15 patients) were discovered. The case distribution included: nodular goitre 6; chronic lymphocytic thyroiditis (C.L.T.) 3 cases; Riedel's thyroiditis l; follicular adenoma l; non-toxic hyperplasia (pseudo-follicular adenoma) 3 and papillary carcinoma 2.

The follicular problems will be discussed in detail. From this modest sized series significant clinical, operative and cyto-histological difficulty was recorded in 10/16 (62%) of patients. The cytopathology of CLT, non-toxic hyperplasia of childhood and the papillary neoplasms was significant and requires emphasis.

Conclusions are tentative. They are:

(1) The valuable status of fine needle aspiration cytology - under a brief general anaesthesia if necessary - for all goitres.
(2) A need for greater recognition of the prevalence of C.L.T. in this age group.
(3) The value of per-operative frozen section diagnosis in neoplastic cases to influence the extent of surgery.
(4) The exacting 'borderline diagnosis' between non-toxic hyperplasia and follicular adenoma.

**REFERENCES**

1. P. Fister-Goedeke L, Stauffer U. G. (1983). Thyroid carcinoma in childhood. In: Rickham PP, Hecker W Ch, Prevot J: Eds. Endocrine Disorders and Tumours in Children. Baltimore Munich: Urban Schwarzenberg .
2. Siegal A., Mimouni M., Kovalivker M. *et al.* (1983). Latent childhood thyroid carcinoma in diffuse lymphocyte thyroiditis. *J. Surg. Oncology*: **23**: 155-157.

### THE MYTH OF THE SMALL DISTRICT GENERAL HOSPITAL

R. Kulkarni and D. A. Griffiths
East Somerset NHS Trust Hospital, Yeovil , Somerset

The ever increasing trend towards larger surgical centres with sub-specialisation has recently been encouraged by Politicians, Managers and Royal Colleges.

In practice, the adequately staffed District General Hospital Surgical Department can deal with the majority of cases without referral to Tertiary centres.

In East Somerset, where over 25% of all the Family Doctor referrals are to the General Surgeons, Clinical Audit has confirmed less than one handful were sent elsewhere.

At present the main reasons for transfer are for expensive high technology therapies which could be provided on a visiting mobile basis for the District Hospitals. Only one person out of 4,800 patients studied was sent elsewhere for diagnosis. The correct provision of on site surgical resources with visiting treatment pantechnovans removes the need for large regional centres and provides an equitable distribution of quality Health Care.

### LOCAL RECURRENCE AFTER LOCALLY CURATIVE COLORECTAL CANCER RESECTION

H. Thompson

Gloucestershire Royal Hospital

The published big differences in rates of local recurrence after locally curative resection of colorectal cancer need explaining. If like is being compared with like and the reports are correct, then some surgeons are more successful in eradicating the disease from its primary site than others. One possible source of trouble is exfoliated colorectal cancer cells, which have been shown capable of implantation and growth. Another is failure, in the case of rectal carcinoma, to excise the entire mesorectum.

To illustrate management of both threats the outcome in all 107 cases of rectal cancer treated by locally curative resection between 1985 and 1991 is presented. There were only three local recurrences but even so it is argued that each could have been prevented.

The study suggests that in all except locally palliative resections (when the primary is peeled or finger fractured from irremovable adjacent structures and regrowth would be inevitable) local recurrence after locally curative procedures is an avoidable tragedy. Whether the technique of on-table colonic lavage used by the author to eliminate exfoliated cells in addition to the standard precautions, is necessary to achieve the aim, remains open to debate.

### STARTING FEMOROTIBIAL BYPASS IN A DISTRICT HOSPITAL

M. G. Wyatt, L. J. M. T. Tambeur, V. F. M. Kernick, H. Clark, W. B. Campbell
Royal Devon and Exeter Hospital.

Femorotibial bypass is not yet done frequently in many district hospitals, because it is time consuming and the risk of failure is substantial, especially during the learning curve. We have reviewed the early results of a single Consultant Surgeon, and his team.

During 1987-91, 75 femorotibial grafts were performed in 66 patients (43 male, aged 45-93 median 73 years). Indications were ulceration or gangrene in 50, rest pain alone in 17 and severe claudication in 8 cases. Fifty seven grafts were autogenous vein, 14 P.T.F.E., 2 composite, and 2 umbilical vein.

Overall, 19 grafts (25.3%) failed within the first 30 days (2 others were salvaged after occlusion) and 20 amputations were required (5 despite patent grafts). There were 2 early deaths (mortality 2.7%).

At the end of the five year period a total of 37 patients had required amputation (0-21 months, median 1 month after operation), and 18 had died. Nineteen (40% of survivors) were alive with patent grafts.

These disappointing early results were due to an initial technical learning curve, after which increased confidence led to reconstructing patients with inadequate distal arteries. Latterly, a more selective approach, with extension of operating time to revise imperfect results as required, has produced improved graft patency. Limb salvage can be achieved in a worthwhile proportion of these patients.

### IS REGULAR OUTPATIENT FOLLOWUP NECESSARY AFTER ARTERIAL RECONSTRUCTION?

T. Elliott, J. M. Dunn, J. Lavy, A. Bell, V. F. M. Kernick
W. B. Campbell.
Royal Devon and Exeter Hospital.

By tradition patients have been reviewed regularly after arterial reconstruction, but Harris[1] has suggested that they should be seen only once unless graft surveillance by scanning is indicated. We have always done this, and reviewed a cohort of patients for the effectiveness and acceptability minimal clinic followup.

Patients who had grafts for lower limb ischaemia during 1987-9 were reviewed. There were 173 (116 male: aged 40-95 years, median 71) of whom 48 had proximal and 131 infrainguinal procedures (some had both), prior to introduction of duplex graft surveillance. During the followup period of 30-66 months 100 patients had died or required amputation. The remaining 73 were invited for review by examination and structured questionnaire, and 64 (87%) attended.

These patients had a median of 1 postoperative review (range 0-6)which 68% found helpful for reassurance, and only 26% would have preferred another. They had been told to report immediately if concerned, and 27 (42%) had done so. Fourteen (22%) had reoperations. 80% grafts remained patent.

Minimal outpatient followup is acceptable to most patients, and most present if symptoms of graft occlusion develop, provided they have been told to do so. Further routine review should be restricted to appropriate vascular laboratory surveillance of selected grafts.

### REFERENCE

1. Harris P. L. (1991). Follow-up after reconstructive arterial surgery. *Eur. J. Vasc. Surg.*: **5**: 369-73.

Bristol Royal Infirmary

